# Daytime Dependence of the Activity of the Rat Brain Pyruvate Dehydrogenase Corresponds to the Mitochondrial Sirtuin 3 Level and Acetylation of Brain Proteins, All Regulated by Thiamine Administration Decreasing Phosphorylation of PDHA Ser293

**DOI:** 10.3390/ijms22158006

**Published:** 2021-07-27

**Authors:** Vasily A. Aleshin, Artem V. Artiukhov, Thilo Kaehne, Anastasia V. Graf, Victoria I. Bunik

**Affiliations:** 1 A.N. Belozersky Institute of Physicochemical Biology, Lomonosov Moscow State University, 119991 Moscow, Russia; aleshinvasily@gmail.com (V.A.A.); whitelord_1994@mail.ru (A.V.A.); nastjushka@gmail.com (A.V.G.); 2 Faculty of Bioengineering and Bioinformatics, Lomonosov Moscow State University, 119991 Moscow, Russia; 3 Institute of Experimental Internal Medicine, Otto-von-Guericke University, D-39120 Magdeburg, Germany; kaehne@med.ovgu.de; 4 Faculty of Nano-, Bio-, Informational, Cognitive and Socio-Humanistic Sciences and Technologies at Moscow Institute of Physics and Technology, Maximova Street 4, 123098 Moscow, Russia; 5 Faculty of Biology, Lomonosov Moscow State University, 119234 Moscow, Russia; 6 Department of Biochemistry, Sechenov University, Trubetskaya, 8, bld. 2, 119991 Moscow, Russia

**Keywords:** brain proteins acetylation, diurnal regulation, pyruvate dehydrogenase, PDHA phosphorylation, SIRT2, SIRT3, thiamine diphosphate, thiol-disulfide metabolism

## Abstract

Coupling glycolysis and mitochondrial tricarboxylic acid cycle, pyruvate dehydrogenase (PDH) complex (PDHC) is highly responsive to cellular demands through multiple mechanisms, including PDH phosphorylation. PDHC also produces acetyl-CoA for protein acetylation involved in circadian regulation of metabolism. Thiamine (vitamin B1) diphosphate (ThDP) is known to activate PDH as both coenzyme and inhibitor of the PDH inactivating kinases. Molecular mechanisms integrating the function of thiamine-dependent PDHC into general redox metabolism, underlie physiological fitness of a cell or an organism. Here, we characterize the daytime- and thiamine-dependent changes in the rat brain PDHC function, expression and phosphorylation, assessing their impact on protein acetylation and metabolic regulation. Morning administration of thiamine significantly downregulates both the PDH phosphorylation at Ser293 and SIRT3 protein level, the effects not observed upon the evening administration. This action of thiamine nullifies the daytime-dependent changes in the brain PDHC activity and mitochondrial acetylation, inducing diurnal difference in the cytosolic acetylation and acetylation of total brain proteins. Screening the daytime dependence of central metabolic enzymes and proteins of thiol/disulfide metabolism reveals that thiamine also cancels daily changes in the malate dehydrogenase activity, opposite to those of the PDHC activity. Correlation analysis indicates that thiamine abrogates the strong positive correlation between the total acetylation of the brain proteins and PDHC function. Simultaneously, thiamine heightens interplay between the expression of PDHC components and total acetylation or SIRT2 protein level. These thiamine effects on the brain acetylation system change metabolic impact of acetylation. The changes are exemplified by the thiamine enhancement of the SIRT2 correlations with metabolic enzymes and proteins of thiol-disulfide metabolism. Thus, we show the daytime- and thiamine-dependent changes in the function and phosphorylation of brain PDHC, contributing to regulation of the brain acetylation system and redox metabolism. The daytime-dependent action of thiamine on PDHC and SIRT3 may be of therapeutic significance in correcting perturbed diurnal regulation.

## 1. Introduction

The brain energy metabolism strongly depends on glucose oxidation, with the mitochondrial pyruvate dehydrogenase (PDH) multienzyme complex (PDHC) playing a key regulatory role in coupling the glycolytic and mitochondrial parts of the glucose oxidation pathway. Perturbations in this coupling occur in different pathologies linked to essential metabolic transformations, such as occurring in malignancies or diabetes. Apart from PDH, PDHC includes the second (dihydrolipoyl acetyl transferase, DLAT) and third (dihydrolipoyl dehydrogenase, DLD) catalytic components, with DLAT binding PDH kinases stronger than PDH phosphatases [1]. Isoenzymes of PDH kinases (PDK1–4) and phosphatases (PDP1–2) determine phosphorylation status of PDHA (alpha subunit of PDH component of the complex), controlling the PDHC activity. The phosphorylation of PDHA at Ser293, localized to the PDH substrate channel, and two other well-characterized sites, Ser300 and Ser232, inactivates PDH, with the most pronounced effect imposed by the phosphorylation of Ser293 [1,2]. PDK2 preferentially phosphorylates Ser293 [1], while the PDH phosphatases are more active at the other sites [1,3]. In the brain, PDK2 and PDP1 are the most important and abundant players in the phosphorylation-dependent regulation of PDH [4].

In addition to the crucial role in metabolism of glucose, PDHC is an important producer of acetyl-CoA for protein acetylation, with not only mitochondria, but also nucleus comprising PDHC for this purpose [5,6]. In accordance with the acetylation-dependent circadian regulation of central metabolism and its tissue specificity [7,8,9], circadian oscillations of PDK4 have been observed in human peripheral blood mononuclear cells and adipose tissue [10]. Ca^2+^-regulated PDH phosphorylation is associated with rhythmical oscillation of mitochondrial respiration in synchronized hepatic cells [11], although diurnal oscillations of PDHC function have not been noticed in the global proteomics study of mouse liver [12]. In the brain, diurnal regulation of PDHC function is not characterized, despite its utmost importance in view of the brain dependence on oxidative glucose metabolism and the brain-specific regulation of metabolic enzymes by acetylation, different from that in peripheral tissues [8,13]. Presumably, molecular mechanisms of the acetylation-dependent metabolic regulation differ according to its different functional role in the brain that generates circadian signals, and the liver, where circadian changes of metabolism are linked to feeding cycles and most studied, not the least because of their strong manifestations [13,14,15,16]. Moreover, the central position of PDHC in the energy metabolism known to oscillate during the day [10,12], suggests dysregulation of this complex to contribute to multiple pathological states linked to perturbation of circadian rhythms [17,18]. In particular, diminished flux through PDHC may be responsible for the decreased longevity upon overexpression of lactate dehydrogenase (LDH) in adult neurons and glia [19]. Remarkably, pan-neuronal LDH overexpression disrupts circadian rhythms in locomotion and accelerates neurodegeneration, while the LDH downregulation has opposite effects [19].

The goal of our current work is to characterize the daytime dependence of PDHC function in the brain, molecular mechanisms of this regulation, its relevance for acetylation of the brain proteins and metabolic regulation, and potential involvement of thiamine (vitamin B1) in the regulation. Thiamine is a precursor of the PDH coenzyme, thiamine diphosphate (ThDP), that is the major component of thiamine pool in most tissues [20]. In addition to this coenzyme role, thiamine and its derivatives regulate multiple protein targets by non-coenzyme binding which is observed not only among the enzymes metabolically linked to PDHC [13,21,22], but also among general regulators of oxidative metabolism, such as transcriptional factor p53 and PDH kinases [21,23,24,25,26,27]. In particular, ThDP may activate PDHC function directly (as a coenzyme), and indirectly (as an inhibitor of PDH kinases). The ThDP interaction with p53 and PDH kinases is important for the anticancer action of thiamine and its derivatives [23,27,28].

In this work, we assess the daytime dependence of the brain PDHC function, phosphorylation, expression of catalytic, and regulatory components, combining this analysis with quantification of the brain protein acetylation and its regulators, such as the mitochondrial sirtuin 3 (SIRT3) and the brain-abundant sirtuin 2 (SIRT2). We show the daily changes in the brain PDHC activity, their abolition by thiamine administration which also reduces phosphorylation of the PDHA Ser293 and the levels of SIRT3 in the morning. Significant metabolic impact of this regulation of PDHC activity by thiamine administration is demonstrated by correlation analysis, exposing the network changes in the relationships between the PDHC structure, function, and role in brain protein acetylation.

## 2. Results

### 2.1. Thiamine Abrogates the Daytime-Dependent Changes in the Brain PDH Activity and Decreases Ser293 Phosphorylation of PDHA1

**Figure 1:**
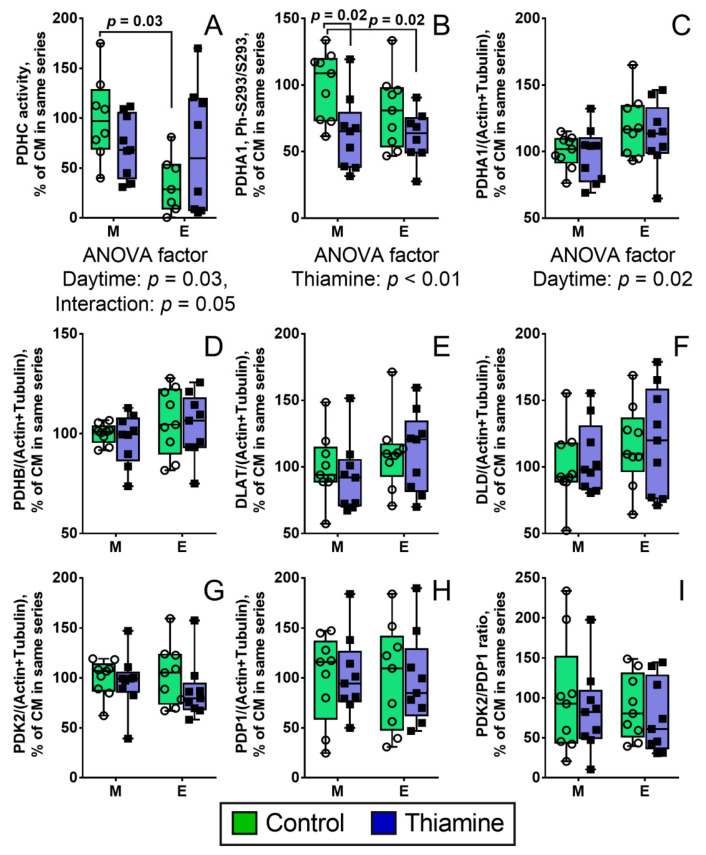
Daytime- and thiamine-dependent regulation of the PDHC activity (**A**); PDHA1 Ser293 phosphorylation (**B**); and protein levels of the catalytic and regulatory components of PDHC (**C**–**I**); The protein components are shown as UNIPROT gene names on the Y axes. The data are presented as box-plots with values determined in individual animals and their median. M—morning, E—evening. Each group is comprised of nine animals, n = 9 (**B**–**I**); with negative values of the PDHC activity being excluded (especially in Control Evening group, see Methods), n = 7–9 (**A**). The data are analyzed by two-way ANOVA with Tukey post-hoc test. *p*-values of significant differences between the four studied groups are indicated on the graphs. In case of significance (*p* ≤ 0.05), *p*-values for influence of the analyzed factors (daytime and thiamine) and their interaction are indicated under the graphs.

PDHC assays in homogenates of the rat cerebral cortices reveal diurnal regulation of the complex in control animals (Figure 1A). Evenings, the PDHC activity is three-fold lower, compared to mornings (*p* = 0.03). This difference is no more observed after rats are supplemented with a high dose of thiamine (Figure 1A). ANOVA points to a significant (*p* = 0.05) interaction between the ‘daytime’ and ‘thiamine’ factors (Figure 1A), correspondent to different directions of the minor thiamine effects on PDHC activity in the morning (a decrease) or evening (an increase). Simultaneously, thiamine administration in the morning significantly decreases the level of phosphorylated Ser293 of PDHA1 (the brain isoenzyme alpha subunit), compared to the control rats (*p* = 0.02), with the effect not observed upon the evening administration of thiamine (Figure 1A). The phosphorylation of PDHA at sites other than Ser293 has not been detectable in analyses of the brain homogenates.

Comparison of PDHA1 protein levels in the paired (with and without thiamine) groups of evening and morning animals reveals a 20% increase of PDHA1 expression in the evening (*p* = 0.02 for the daytime significance by ANOVA) (Figure 1C). This is the only daytime-dependent difference in the protein levels of the analyzed PDHC components, including PDH kinase PDK2 and phosphatase PDP1, in the brain (Figure 1C–H). Thiamine administration has no effect on the protein levels of the PDHC components (Figure 1C–H). The ratio of the PDK2 to PDP1 levels is not altered by daytime or thiamine administration (Figure 1I). 

Thus, the brain PDHC activity reveals diurnal changes, which are nullified by the thiamine administration to animals. Among the PDHC catalytic and regulatory components, only PDHA1 subunit demonstrates significant diurnal differences in its expression (independent of thiamine) and Ser293 phosphorylation (affected by thiamine).

### 2.2. Diurnal Effect of Thiamine Administration on Protein Acetylation System in the Brain

**Figure 2:**
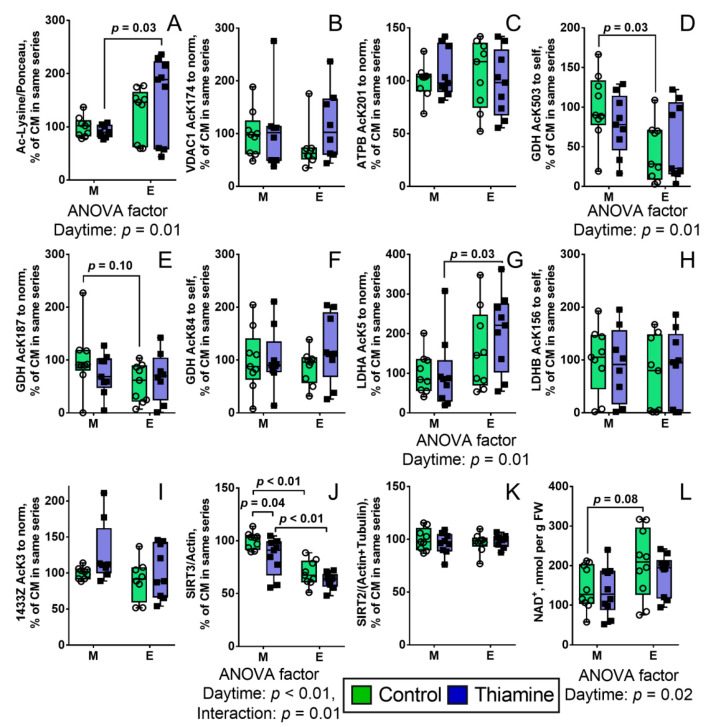
Daytime- and thiamine-dependent assays of protein acetylation in the rat brain homogenates. The graphs show the levels of total protein acetylation (**A**), mitochondrial proteins: VDAC1, acetylation of Lys174 (**B**), ATPB, acetylation of Lys201 (**C**), GDH, acetylation of Lys503 (**D**), GDH, acetylation of Lys187 (**E**), GDH, acetylation of Lys84 (**F**), cytoplasmic proteins: LDHA, acetylation of Lys5 (**G**), LDHB, acetylation of Lys156 (**H**), 1433Z (gene *YWHAZ*), acetylation of Lys3 (**I**) and levels of deacetylases sirtuin 3 (**J**) and sirtuin 2 (**K**) and their substrate NAD^+^ (**L**). The lysine residues in figure legends are referred using single letter code—K. The protein components are shown as UNIPROT gene names on the Y axes, with the exception of GDH (gene *GLUD1*). The data are presented as box-plots with values determined in individual animals and their median. M—morning, E—evening. Number of animals in each of the four groups: n = 9 (**A**–**K**), n = 10 (**L**) (see Methods). The data are analyzed by two-way ANOVA with Tukey post-hoc test. *p*-values of significant differences and also trends (0.1 < *p* < 0.05) between the four studied groups are indicated on the graphs. In case of significance (*p* ≤ 0.05), *p*-values for influence of the analyzed factors (daytime and thiamine) and their interaction are indicated under the graphs.

PDHC is an important producer of acetyl-CoA which is used for post-translational acetylation of proteins, regulating their activities corresponding to diurnal rhythms. Hence, we aimed to reveal how the daily changes of PDHC activity (Figure 1A) would affect the brain acetylation system. As seen from Figure 2A, the total protein acetylation in cerebral cortex, assayed by Western blot, does not show the diurnal changes, but the changes appear after the thiamine administration, that nullifies diurnal difference in PDHC activity (Figure 1A). Such induction of diurnal differences in protein acetylation upon thiamine administration points to the coupling between the PDHC activity (Figure 1A) and acetylation (Figure 2A): The latter is stabilized with the diurnal changes of the former, while the thiamine-induced cancellation of the PDHC diurnal changes causes the changes in the total acetylation.

Total protein acetylation in the brain as revealed by Western blot, is characterized by a major wide band of app. 50 kDa, probably reflecting high acetylation level of tubulin. In view of the high immunoreactivity of this band, the less acetylated proteins of other molecular mass may be not detectable. Therefore, to estimate the protein acetylation we employed an earlier developed approach [13] for mass-spectrometric quantifications of acetylation of particular proteins whose acetylated peptides are well-detectable in the brain homogenates. This high-resolution approach also allows one to quantify acetylation at proteins belonging to different cellular compartments. Acetylation of the mitochondrial proteins has been estimated using acetylatable peptides of porin VDAC1 with Lys174, ATPase subunit ATPB with Lys201, GDH with Lys503, Lys187 and Lys84. Acetylation of cytoplasmic proteins has been assessed using acetylatable peptides of lactate dehydrogenases LDHA with Lys5 and LDHB with Lys156, and the 14-3-3 protein zeta Lys3 (gene *YWHAZ*) (Figure 2B–I). The peptides used for these quantifications are listed in Supplementary Table S1. In the set of selected mitochondrial and cytoplasmic proteins of control rats, the mitochondrial representative GDH exhibits statistically significant (*p* = 0.03) diurnal pattern of acetylation at Lys503 residue (Figure 2D), while none of the cytosolic proteins shows this pattern. In contrast, in the thiamine-treated rats, diurnal changes in acetylation are significant (*p* = 0.03) for the LDHA Lys5 residue (Figure 2G), but disappear in the mitochondrial GDH Lys503 (Figure 2D). The observed pattern of acetylation of the cytosolic protein LDHA is similar to that of the total protein acetylation, presumably dominated by cytosolic tubulins, as noted above. Thus, the diurnal changes in GDH Lys503 acetylation, LDHA Lys5 acetylation and total protein acetylation correspond to the changes in PDHC activity. However, acetylation of mitochondrial protein GDH coincides with the activity, i.e., diurnal changes of GDH acetylation occur or disappear along with those of PDHC activity. In contrast, acetylation of a cytosolic protein LDHA and acetylation of total proteins change with the daytime only when the PDHC activity does not show the diurnal change, i.e., in the thiamine-treated rats (Figure 2D,G vs. Figure 1A and Figure 2A). 

Protein acetylation is strongly regulated by NAD-dependent deacetylases sirtuins, with SIRT2 and SIRT3 being the main cerebral protein deacetylases of cytosolic and mitochondrial proteins, respectively. Our analysis reveals the diurnal changes in the levels of SIRT3 in the rat brain, while the level of SIRT2 remains constant (Figure 2J,K). As can be seen from Figure 2J, SIRT3 level is ~30% lower (*p* < 0.01) evenings than mornings, independently of thiamine administration. Administration of thiamine decreases (*p* = 0.04) SIRT3 level in the morning, but not in the evening, which corresponds to the interaction (*p* = 0.01) of the two factors indicated by ANOVA (Figure 2J). Nevertheless, the diurnal difference in SIRT3 level is preserved after the administration of thiamine.

The levels of cerebral NAD^+^, the substrate of sirtuins, also reveal a trend (*p* = 0.08) to diurnal changes in control rats, exhibiting a higher level of NAD^+^ in the evenings than mornings, while in the thiamine-treated rats, the difference disappears (Figure 2L). 

Thus, major components of the brain mitochondrial acetylation system—including PDHC, NAD^+^, and sirtuin 3—along with a mitochondrial marker of such acetylation, GDH, exhibit coupling in their diurnal changes and regulation of such changes by thiamine administration. Acetylation of the cytosolic protein LDH exhibits a pattern, different from that of the mitochondrial acetylation. In control animals, the cytosolic acetylation does not differ when the changes in mitochondrial acetylation are observed, while similar levels of the mitochondrial acetylation are observed when cytosolic acetylation does differ. 

### 2.3. Correlation Analysis of Coupled Changes in the PDHC Structural and Functional Parameters and Components of the Brain Proteins Acetylation System

Correlations between the parameters characterizing PDHC structure and function, including its activity, Ser293 phosphorylation and protein levels of its components, and parameters of brain proteins acetylation system are shown in Table 1. These correlations characterize ability of animals to support homeostasis by co-adapting individual differences in specific components of the homeostatic network. For instance, strong positive correlations between the expression of the two subunits of PDH—i.e., PDHA1 and PDHB—are observed regardless of thiamine administration, pointing to co-expression of these components at a constant ratio for optimal build-up of the heterodimeric protein. The level of Ser293 phosphorylation positively correlates with PDHA1, PDHB, and PDK2, manifesting a co-adjustment of the phosphorylation level to expression of PDH components and PDK2, that may stabilize the level of PDHC function according to the individual metabolic networks differing in the protein expression. Remarkably, with the strong co-expression of DLAT and DLD (*p* < 0.01), these components do not correlate to PDHA1 and PDHB. This interdependence pattern is in accordance with existence of specific mechanisms of regulation of the starting and rate-limiting PDH component, such as phosphorylation, contributing to the regulation of the PDHC function more than just co-expression of PDH with the other complex components. Remarkably, in the thiamine-treated animals, the interdependence between the PDHC phosphorylation and components expression is shifted to the co-expression of all the PDHC components (Table 1), in accordance with the thiamine effect on the PDHA1 phosphorylation (Figure 1B). At the same time, thiamine increases the number of positive correlations of all the PDHC components with SIRT2, simultaneously decreasing the number of negative correlations between SIRT2 and proteins acetylation (Table 1). All together, these results confirm the thiamine-dependent regulation of interdependence of PDHC function and acetylation, discussed in the previous section. This is further supported by positive correlations of the PDHC activity with acetylation of mitochondrial VDAC1, GDH, or total brain proteins, which disappear after the thiamine treatment (Table 1). Remarkably, acetylation of cytosolic proteins does not correlate with PDHC activity in control animals, or even shows a negative correlation (LDHA) with the activity after the thiamine treatment.

Thus, correlation analysis provides further insights on significance and interdependence of PDHC function with the brain protein acetylation.

### 2.4. Daytime- and Thiamine-Dependent Assays of Enzymes of the PDHC- and Redox-Linked Pathways

**Figure 3:**
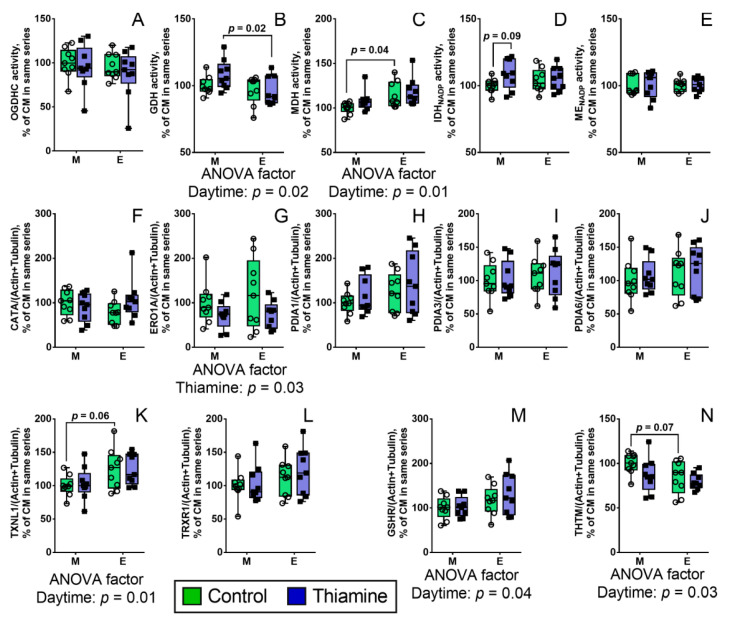
Daytime- and thiamine-dependent assays of the activities or expression of the enzymes of central metabolism linked to PDHC (**A**–**E**) and redox pathways (**F**–**N**). The enzymes and proteins are indicated on the Y axes as the first part of their UNIPROT Entry name (formerly UNIPROT ID). The data are presented as box-plots with median and all points. A single point represents the data from a single rat. M—morning, E—evening. Each group comprises of 9 animals, n = 9. The data were analyzed by two-way ANOVA with Tukey post-hoc test. *p*-values of the significant (*p* ≤ 0.05) ANOVA factors (daytime and thiamine) and their interaction are indicated on the graphs. *p*-values of significant differences between groups and also trends (0.1 < *p* < 0.05) are shown above the corresponding arches.

Circadian rhythms are linked not only to acetylation, but also to the cellular redox status including that of the glutathione buffer [29,30,31]. Due to the thiamine involvement with acetylation of metabolic proteins, on one hand, and with redox-state-supporting proteins and genes, including those controlling the redox potential of cellular thiols and disulfides, on the other hand [21], we have assessed activities or expression of some enzymes of central metabolism related to PDHC pathway and/or redox metabolism, which may be regulated by protein acetylation and/or thiamine itself (Figure 3).

Remarkably, unlike the activity of PDHC (Figure 1A), activity of ThDP-dependent 2-oxoglutarate dehydrogenase (OGDH) multienzyme complex (OGDHC) is not regulated in a daytime- or thiamine-dependent manner (Figure 3A), while the acetylated metabolic partner of OGDHC, GDH, demonstrates such regulation, according to the published data [13]. However, MDH activity shows diurnal changes opposite to those of PDHC activity, which do not respond to thiamine (Figure 3C). As MDH is a metabolic partner of PDHC, providing oxalacetate for incorporation of acetyl-CoA into the TCA cycle, our data stress the importance for the brain metabolism of diurnal changes of PDHC and MDH, on one hand, and specific action of thiamine on diurnal regulation of PDHC through PDHA1 phosphorylation. Other brain enzymes related to the pyruvate and 2-oxoglutarate metabolism, do not show significant diurnal changes either with or without thiamine administration.

Among the nine relatively abundant brain enzymes involved in the redox-dependent processes, the three enzymes of the thiol-disulfide metabolism (TXNL1, GSHR, and THTM) show the daytime-dependent expression (according to ANOVA significance of daytime factor), while ERO1A involved in the formation of disulfides in the synthesized proteins, shows the thiamine dependence (Figure 3F–N).

### 2.5. Correlation Analysis of the Metabolic Impact of Diurnal Pattern of the Brain Protein Acetylation

To reveal a broader interconnection between the PDHC-dependent acetylation and metabolic regulation, the correlation analysis has been performed, considering the estimated parameters (Table 2).

First, Table 2 shows that not only PDHC activity, but also OGDHC activity and catalase expression correlate positively with total acetylation of the brain proteins in control rats. After the thiamine administration, neither PDHC nor OGDHC activities correlate with the total protein acetylation, pointing to concerted regulation of the protein acetylation by the substrate flux through the TCA cycle, which is determined by the acetyl-CoA production from pyruvate by PDHC and the rate-limiting role of OGDHC [32]. Activities or expression of OGDHC and IDH show negative correlations with SIRT2 in the control rats, but only positive correlations with SIRT2, and an increased number of such correlations, are observed in the thiamine-treated rats. Thus, thiamine affects the interconnection between the thiol-disulfide metabolism and SIRT2. Interplay of SIRT3 with redox metabolism and its thiamine dependence is observed in the positive correlation between expression of SIRT3 and catalase (Table 2).

Thus, correlations analysis exposes thiamine dependence of metabolic interconnections between the brain acetylation system, including PDHC as acetyl-CoA producer for proteins acetylation and protein deacylases sirtuins, with the TCA cycle and enzymes of the redox-state-related pathways.

## 3. Discussion

In this work, diurnal changes in the activity of PDHC in the rat brain cortex (Figure 1), and their synchronization with those of mitochondrial deacetylase SIRT3 (Figure 2), NAD^+^ and a mitochondrial acetylation marker GDH, are shown for the first time. In addition, we demonstrate that all of these parameters undergo coupled regulation by thiamine administration, decreasing the phosphorylation of PDHA1 subunit of PDH at Ser293 (Figure 1B). It is noteworthy that in vitro the decreased phosphorylation level activates PDH. However, in vivo we observe the opposite change, with the total tissue PDH activity decreasing at decreased level of the enzyme phosphorylation (Figure 1). This finding manifests the complexity of the PDHC regulation in vivo. In fact, apart from phosphorylation, the PDHC activity is also regulated by acetylation of the catalytic and regulatory components of PDHC [33,34,35], potentially contributing to the observed inactivation of PDHC at decreased level of its phosphorylation (Figure 1). Different saturations of the complex core with the peripheral components may also affect the activity of multienzyme complexes in vivo [1]. Remarkably, the correlation analysis indicates that thiamine changes the correlations between the expression of the catalytic and regulatory components of PDHC, specifically affecting those of PDP1 (Table 1). The strong correlation between the expressions of the PDH kinase (PDK2) and phosphatase (PDP1), present in the control animals, disappears after the thiamine administration. Besides, the second (DLAT) and third (DLD) components of PDHC correlate negatively to the PDHC activity in the control animals, but the activity becomes positively correlated to these components in the thiamine-treated animals. As the second DLAT component of PDHC is involved in the inactivating self-acetylation of the complex, also inherent in the PDH component [34,35], the thiamine-changed interplay between the catalytic and regulatory components of PDHC may well manifest the interactions between the PDH (de)phosphorylation and (de)acetylation, shown in independent work [33]. These interactions may be responsible for the decreased PDHC activity upon dephosphorylation in vivo, compared to the activating action of dephosphorylation in vitro. This result is also important in view of general interpretations of the in vivo results, based on findings in model systems, where only one factor of influence is studied.

Comparison of our data for the rat brain (Figure 1) and published data for the mouse liver [36] reveals the tissue-specific difference in regulation of SIRT3 function by NAD^+^. In the brain, both the NAD^+^ level and SIRT3 expression show coupled changes during the day, although the former varies less significant than the latter (Figure 2). In contrast, in the liver mitochondria the SIRT3-dependent acetylation of target proteins is regulated by the oscillations of NAD^+^ level, which are more significant than those of SIRT3 [36].

Finally, our analysis has also revealed a remarkable interplay between acetylation of the mitochondrial and cytosolic proteins. Acetylation of the former, represented by GDH, reproduces the diurnal changes in activity of PDHC, while acetylation of the latter responds not to the diurnal changes of PDHC activity, but to their perturbations in the thiamine-treated animals. This finding may be related to an earlier suggestion that thiamine may regulate the distribution of acetyl-CoA between the mitochondria and cytosol ([21] and references therein). This is further supported by the thiamine-induced enhancement of the correlations between the mainly cytosolic and abundant in the brain SIRT2 and acetylation of mitochondrial proteins (Figure 2, Table 2). Commonly, SIRT2 is considered as the cytosolic protein, with the tubulin shown as its main target [37]. The role of SIRT2 in the alpha-tubulin deacetylation may be involved in neuropathologies, such as Parkinson’s disease or Alzheimer’s disease. In fact, inhibition or knockout of SIRT2 in the molecular models of these diseases promoted alpha-tubulin acetylation, trafficking of misfolded proteins and autophagy, simultaneously restoring mitochondrial function [38,39]. However, regulation of mitochondrial acetylation by SIRT2 may also involve the direct action on mitochondrial proteins, as association of SIRT2 with the inner mitochondrial membrane is shown in the mouse brain by immunohistochemistry [40].

The role of proteins of cellular redox homeostasis in cellular timekeeping is emerging [41], supported by the daytime-dependence of thioredoxin-like protein 1 (TXNL1), glutathione reductase (GSHR) and mercaptopyruvate sulfurtransferase (THTM) protein, shown in our work. In view of involvement of sirtuins in circadian rhythms [42], the thiamine-dependent correlations between expression of SIRT2 and expression of proteins of the thiol/disulfide metabolism—such as ERO1A, THTM (in the brain of both control and thiamine-treated rats); and PDIA3, PDIA6, GSHR, and TRXR1 (in the brain of thiamine-treated rats) (Table 2)—are in good accord with the metabolic significance of the interplay between the protein acetylation and disulfide bonds formation, exemplified, in particular, by the regulation of SIRT2 through S-glutathionylation [43].

Remarkably, ERO1A is a prognostic marker and a promising target in different types of cancer, such as non-small cell lung cancers, osteosarcoma, pancreatic cancer [44,45,46]. ERO1A expression is associated with shorter overall survival in NSCLC patients [44]. In pancreatic cancer cells, signaling through ERO1A is involved with metabolic reprogramming to glycolysis [46], which reduces the flux through PDHC. The recent data on the ThDP-mediated antiproliferative effect in a NSCLC cell line A549 [28] coupled to metabolic reprogramming of these cells [47], may therefore involve also ERO1A, whose expression is decreased by thiamine, as shown in our current work. 

The thiamine-dependent downregulation of the ERO1A level, observed independently of daytime (Figure 3), is of particular interest also in view of neurodegeneration-causing events, which are stimulated by thiamine deficiency [48] and decreased by thiamine addition [22,49,50,51]. ERO1A is a FAD-dependent protein, which reoxidizes protein disulfide isomerases (such as PDIA1) and produces ROS (hydrogen peroxide), being involved in endoplasmic reticulum stress [44,52]. In its turn, endoplasmic reticulum stress is a component of neurodegenerative diseases, linked to autophagy [53] and perturbed calcium exchange between endoplasmic reticulum and mitochondria [54]. Inhibiting the ERO1A pathway by either pharmacological or genetic means [45,46] reduces ROS production upon endoplasmic reticulum stress. Positive action of thiamine on metabolic adaptations and glutathione redox status in injured neural tissues [22,49,50] is thus in good agreement not only with the general metabolic action of thiamine as activator of oxidative glucose metabolism, but also with the thiamine-dependent downregulation of ERO1A in the rat brain shown in our current study (Figure 3). These complex effects may contribute to beneficial action of pharmacological forms of thiamine in patients with neurodegenerative diseases [51]. Regarding this beneficial action, it is also worth noting that, according to the data of our current study (Table 2), thiamine administration decreases the interdependence between the proteins constituting PDHC and related to metabolism of thiols and disulfides, and components of the brain acetylation system. Strong correlations between all these components of metabolic network in the normal rat brain, observed in our study, testify to their intimate interplay which is subject to regulation by thiamine administration. 

## 4. Materials and Methods

### 4.1. Materials

When not specified otherwise, chemicals were obtained from Sigma-Aldrich (Helicon, Moscow, Russia). NAD^+^ was purchased from GERBU (Biolab-Ltd., Moscow, Russia). Formate dehydrogenase for NAD^+^ assays was obtained from Federal Research Center of Biotechnology/Innotech MSU (Moscow, Russia). Thiamine*HCl was from SERVA Electrophoresis GmbH (Helicon, Moscow, Russia). Deionized MQ-grade water was used for solution preparations. 

### 4.2. Animal Experiments and Thiamine Administration

All animal experiments were performed according to the Guide for the Care and Use of Laboratory Animals published by the European Union Directives 86/609/EEC and 2010/63/EU, and were approved by Bioethics Committee of Lomonosov Moscow State University (protocol number 69-o from 09.06.2016) as described previously [13].

Briefly, 36 Wistar (RRID: RGD_13508588) male rats (approx. 3–4 months and 290–320 g) were used in three independent animal series by 12 rats, randomly divided into four subgroups by three animals. Thiamine (pH = 7) was administered in the morning (ZT 2 ± 1) or in the evening (ZT 9 ± 1) at the dose of 400 mg of thiamine per kg as a single intraperitoneal injection. The control groups of animals received a similar injection of physiological solution (0.9% sodium chloride). Animals were subjected to physiological monitoring, and killed by decapitation 24 h after injections. The brains were excised and transferred on ice, where the cortices were separated to be frozen in liquid nitrogen 60–90 s after decapitation. The cortices were stored at −70 °C. NAD^+^ assays were performed in similar series employing 40 rats. The choices of the used time intervals and thiamine dose were justified before [13].

### 4.3. Activity Assays

Enzyme activities were measured in the cerebral cortex homogenates, prepared at the day of analysis as described before [13]. The activities of PDHC, OGDHC, GDH, MDH, NADP-dependent IDH and ME were assayed spectrophotometrically using Sunrise plate reader (Tecan, Austria) by measuring initial rates of the reactions as described previously [55]. Activity was expressed as μmol of product formed per min per g of the tissue fresh weight (FW). When the negative slopes were obtained after subtracting background from the reaction rate (4 out of 36 PDHC assays distributed over control morning (n = 1), control evening (n = 2) and thiamine evening (n = 1) groups), they were excluded from the analyses rather than nullified, as the artificial increase of a negative value to zero could introduce a bias, not present in other values.

### 4.4. Analysis of the Peptides by LC-MS/MS

The polyacrylamide gel areas between the 25 kDa and 75 kDa, corresponding to most of the proteins of cerebral cortex homogenates, including all the components of PDHC, were excised and subjected to in-gel digestion and Nano-LC-MS/MS analysis as described earlier [13,21]. The spectra acquisition consisted of an orbitrap full MS scan (FTMS; resolution 60,000; *m*/*z* range 400–2000) followed by up to 15 LTQ MS/MS experiments (Linear Trap; minimum signal threshold: 500; dynamic exclusion time setting: 30 s; singly charged ions were excluded from selection, normalized collision energy: 35%; activation time: 10 ms). Raw data processing, protein identification and phosphorylation assignment of the high resolution orbitrap data were performed by PEAKS Studio 8.0 (Bioinformatics Solutions, Waterloo, ON, USA). False discovery rate was set to <1%.

### 4.5. Quantification of the Protein Levels, PDHA1 Ser293 Phosphorylation, and Acetylation of Other Proteins

MS-based peptide relative quantification of PDHC components PDHA1 (somatic), PDHB, DLAT, DLD, PDK2, PDP1 together with SIRT2, beta-actin (ACTB), tubulin beta-3 (TBB3) peptides and peptides of redox proteins: catalase (CATA), mercaptopyruvate sulfurtransferase (THTM or MPST), endoplasmic reticulum oxidoreductase 1 alpha (ERO1A), prolyl 4-hydroxylase subunit beta (PDIA1 or P4HB) with its paralogs PDIA3 and PDIA6, glutathione-disulfide reductase (GSHR or GSR), thioredoxin reductase 1 (TRXR1 or TXNRD1) and thioredoxin like 1 (TXNL1), presented in the Supplementary Table S1, was performed using the Skyline platform [56] as described previously [13]. Briefly, the relative protein expression levels in each sample were calculated using the sum of unique peptides, corresponding to a particular rat protein (Supplementary Table S1). Relative expression of the detected proteins was normalized to the sum of relative expressions of beta-actin and tubulin beta-3 (Supplementary Table S1)—separately within each sample corresponding to a single animal. The length, residue numbering, and known phosphorylatable somatic PDHA1 residues are identical between the human and rat sequences (98% sequence identity). The relative level of PDHA1 Ser293 phosphorylation was determined as the ration of the two peptides, comprising the phosphorylated and dephosphorylated Ser293 residue, correspondingly (Supplementary Figure S1). The levels of acetylation of VDAC1 Lys174, ATPB Lys201, GDH Lys503, Lys187, Lys84, LDHA Lys5, LDHB Lys156, and 1433Z Lys3 (gene *YWHAZ*) were quantified as described previously [13]. Normalization was done either to the peptide, containing the same Lys residue in the deacetylated form or to the sum of normalizing peptides of the same protein, listed in the Supplementary Table S1. All the used peptides are listed in the Supplementary Table S1 together with the corresponding mass and charge parameters.

### 4.6. Assay of Total Lysine Acetylation by Western Blotting

The crude cerebral homogenates, prepared as described before [13], were dissolved 60-fold in the sample buffer (60 mM Tris-HCl pH 6.8, 10% glycerol, 2% SDS, 5% β-mercaptoetanol, 0.01% bromophenol blue) and incubated at 95 ℃ for 5 min. The levels of lysine acetylation in the cerebral homogenates were assayed using anti-acetylated-lysine primary antibody (Cell Signaling Technology, #9441). The Ponceau S solution 0.1% (*w/v*) in 5% acetic acid (Sigma-Aldrich, St. Louis, MO, USA, P7170) was used for the total protein staining and normalization, according to manufacturer’s instructions. Representative images are shown in Supplementary Figure S2. Briefly, after the electrophoresis and transfer of the proteins to the PVDF membrane, the membrane was stained in Ponceau S solution for 5 min, washed, pictured, and destained according to manufacturer’s instructions (Sigma-Aldrich, P7170). Then the membrane was blocked by the 5% BSA solution in TBST for 1 h. The 1:1000 dilution of the primary antibodies and 1:3000 dilution of secondary antibodies (Cell Signaling Technology, #7074) were used.

### 4.7. Assay of SIRT3 by Western Blotting

The levels of sirtuin 3 in the cerebral homogenates were assayed using the corresponding primary antibody (Cell Signaling Technology, Danvers, MD, USA #5490). The beta-actin antibody (Cell Signaling Technology, #8457) was used for the normalization. Both primary antibodies were used at 1:2000 dilution, with the secondary antibodies (Cell Signaling Technology, #7074) used at 1:3000 dilution. Representative images are shown in Supplementary Figure S3.

### 4.8. Preparation of Tissue Extracts and Quantification of NAD^+^ Levels

Methanol/acetic acid extracts of rat cerebral cortices were prepared as described before [57]. Briefly, 0.5 g of brain tissue was homogenized in 4 mL ice-cold methanol, with the resulting homogenate diluted with 0.2% acetic acid at 1:1.5 ratio. After 30 min incubation on ice, the suspension was centrifuged and clear supernatants were stored at −70 °C until analysis. NAD^+^ was measured in the extracts using recombinant formate dehydrogenase [58]. Briefly, 1 mL aliquots of the extracts were vacuum dried and redissolved in 100 µL of 0.1 M Bis-Tris buffer (pH = 6.5). Then 5 or 10 µL of the redissolved extracts were mixed with 190 µL of the reaction medium comprising 0.6 M sodium formate in 0.1 M sodium phosphate buffer, pH 7.0, followed by the addition of app. 0.06 units of formate dehydrogenase (6 mg/mL solution in 0.1 M sodium phosphate buffer, pH 7.0). Fluorescence change after 10 min of the reaction (λex = 340 nm, λem = 465 nm) was detected with CLARIOstar microplate reader (BMG Labtech, Ortenberg, Germany) and used to quantify NAD^+^ in the samples. Calibration curve comprised NAD^+^ standards in the range of 0.01–0.2 nmol/well.

### 4.9. Statistics

Statistical analysis was performed using GraphPad Prism, version 7.0 (GraphPad Software Inc., La Jolla, CA, USA). Comparisons between the four experimental groups were done using two-way ANOVA with post hoc Tukey’s test. The two factors of two-way ANOVA, namely daytime of injections/sample collections and thiamine administration, as well as their interaction, were considered. Normal distribution was tested using D’Agostino and Pearson omnibus normality test (*p* < 0.05). The ROUT test for outliers [59] was applied, resulting in no exclusion of data points. Correlation analysis was made using Pearson correlations after checking normality.

## 5. Conclusions

Our work has revealed that the brain PDHC function, SIRT3 expression, and mitochondrial acetylation decrease evenings compared to mornings. In contrast, expression of the PDHA1 subunit of PDHC, levels of NAD^+^ and malate dehydrogenase activity increase evenings compared to mornings. The daytime-dependent expression of the brain proteins of thiol-disulfide metabolism is shown: expression of thioredoxin-like protein 1 and glutathione reductase increases evenings compared to mornings, while mercaptopyruvate sulfurtransferase shows the opposite change. Morning administration of high doses of thiamine decreases PDHA1 phosphorylation, and SIRT3 protein level. Metabolic interplay between the brain PDHC function and acetylation, along with the regulation of this interplay by thiamine administration, are supported by correlation analysis. Independent of daytime, thiamine decreases expression of the endoplasmic reticulum stress marker ERO1A protein.

## Figures and Tables

**Table 1:** Correlations of the functional and structural status of PDHC with parameters of cerebral acetylation system in the control and thiamine-treated rats. The following parameter sets determined in each animal are used for the correlation analysis: the PDHC activity, relative level of PDHA Ser293 phosphorylation (P-Ser293), relative protein levels of the PDHC components and sirtuins (SIRT2 and SIRT3), relative level of lysine acetylation (AcK) of either total cerebral proteins (determined by Western blot) or particular protein sites (determined by mass spectrometry). In each cell, Pearson’s correlation coefficients and *p*-values are presented above and below, respectively, for the pairs of parameters indicated in the header row and column. The data obtained during the day for the control rats and those which received thiamine, are presented in the table parts below and above the diagonal line, correspondingly. Statistically significant (*p* < 0.05) positive and negative correlations are marked by the red and blue color of the cells, correspondingly. The cluster of correlations between the expression of PDHC components is marked with a bold square. Columns and rows, corresponding to acetylation of mitochondrial proteins, are marked with yellow.

	Thiamine	PDHC Activity	P-Ser293	PDHA1	PDHB	DLAT	DLDH	PDK2	PDP1	SIRT3	SIRT2	AcK Total	AcK174 VDAC1	AcK201 ATPB	AcK503 GDH	AcK187 GDH	AcK84 GDH	AcK5 LDHA	AcK156 LDHB	AcK3 1433Z
Control																				
PDHC activity			−0.13 0.61	−0.06 0.83	0.00 1.00	−0.12 0.65	−0.28 0.26	−0.07 0.78	−0.37 0.14	0.00 0.99	−0.21 0.41	0.20 0.44	0.32 0.21	0.43 0.07	0.34 0.16	0.36 0.14	0.21 0.39	−0.56 0.02	0.18 0.47	0.18 0.50
P-Ser293		−0.45 0.06		0.43 0.08	0.47 0.05	0.56 0.02	0.61 0.01	0.47 0.05	0.37 0.13	0.00 1.00	0.51 0.03	−0.74 0.00	−0.48 0.05	−0.46 0.06	−0.33 0.18	−0.23 0.37	−0.31 0.21	0.06 0.82	0.33 0.19	−0.09 0.75
PDHA1		−0.17 0.49	0.52 0.03		0.88 0.00	0.25 0.32	0.26 0.30	0.88 0.00	0.19 0.44	−0.08 0.75	0.56 0.02	−0.57 0.01	−0.52 0.03	0.23 0.36	−0.26 0.31	0.14 0.58	−0.12 0.63	−0.32 0.19	0.14 0.58	−0.55 0.03
PDHB		−0.31 0.22	0.68 0.00	0.92 0.00		0.27 0.28	0.35 0.15	0.94 0.00	0.30 0.22	0.16 0.54	0.75 0.00	−0.56 0.02	−0.58 0.01	0.16 0.52	−0.39 0.11	0.00 0.99	−0.30 0.22	−0.46 0.06	0.11 0.67	−0.43 0.09
DLAT		−0.50 0.03	0.24 0.34	0.27 0.28	0.32 0.20		0.81 0.00	0.42 0.08	0.55 0.02	0.05 0.84	0.58 0.01	−0.49 0.04	−0.29 0.27	−0.32 0.20	−0.54 0.02	−0.44 0.07	−0.65 0.00	0.09 0.72	0.34 0.17	−0.16 0.55
DLD		−0.51 0.03	0.37 0.13	0.36 0.14	0.39 0.11	0.97 0.00		0.40 0.10	0.76 0.00	0.27 0.28	0.64 0.00	−0.62 0.01	−0.43 0.08	−0.26 0.29	−0.70 0.00	−0.63 0.00	−0.77 0.00	0.14 0.57	0.41 0.09	0.11 0.68
PDK2		−0.17 0.51	0.61 0.01	0.95 0.00	0.94 0.00	0.33 0.18	0.40 0.10		0.37 0.14	0.14 0.59	0.81 0.00	−0.56 0.02	−0.60 0.01	0.07 0.77	−0.36 0.14	−0.06 0.82	−0.31 0.21	−0.38 0.12	0.10 0.70	−0.40 0.13
PDP1		−0.42 0.08	0.36 0.14	0.41 0.09	0.48 0.04	0.87 0.00	0.87 0.00	0.47 0.05		0.42 0.09	0.71 0.00	−0.28 0.25	−0.47 0.06	−0.40 0.10	−0.67 0.00	−0.60 0.01	−0.69 0.00	0.11 0.66	0.27 0.29	0.10 0.71
SIRT3		0.42 0.08	−0.03 0.90	−0.09 0.72	−0.03 0.90	0.04 0.87	−0.05 0.85	0.09 0.73	−0.11 0.68		0.31 0.21	−0.11 0.65	−0.42 0.10	0.08 0.75	−0.17 0.50	−0.39 0.11	−0.20 0.43	−0.25 0.31	0.02 0.95	0.43 0.10
SIRT2		−0.45 0.06	0.76 0.00	0.64 0.00	0.83 0.00	0.31 0.22	0.38 0.12	0.76 0.00	0.44 0.06	0.08 0.75		−0.43 0.07	−0.52 0.03	−0.19 0.45	−0.64 0.00	−0.38 0.11	−0.65 0.00	−0.20 0.41	0.12 0.63	−0.26 0.34
AcK total		0.72 0.00	−0.64 0.00	−0.44 0.07	−0.54 0.02	−0.44 0.07	−0.59 0.01	−0.43 0.07	−0.49 0.04	0.43 0.08	−0.64 0.00		0.73 0.00	0.05 0.84	0.28 0.27	0.08 0.76	0.23 0.35	0.10 0.68	−0.28 0.26	0.15 0.59
AcK174 VDAC1		0.61 0.01	−0.69 0.00	−0.67 0.00	−0.71 0.00	−0.29 0.26	−0.46 0.07	−0.60 0.01	−0.38 0.13	0.37 0.14	−0.70 0.00	0.86 0.00		−0.05 0.85	0.24 0.35	−0.07 0.80	0.12 0.64	−0.01 0.97	−0.15 0.58	0.11 0.69
AcK201 ATPB		0.44 0.07	−0.10 0.68	0.17 0.51	0.13 0.60	−0.10 0.699	−0.17 0.50	0.21 0.40	0.04 0.86	0.22 0.37	0.00 0.99	0.49 0.04	0.31 0.23		0.27 0.28	0.50 0.04	0.29 0.24	−0.27 0.28	0.08 0.75	−0.02 0.93
AcK503 GDH		0.62 0.01	−0.47 0.05	−0.27 0.28	−0.44 0.07	−0.66 0.00	−0.69 0.00	−0.39 0.11	−0.79 0.00	0.15 0.56	−0.67 0.00	0.61 0.01	0.47 0.05	0.23 0.36		0.68 0.00	0.94 0.00	0.05 0.85	−0.13 0.60	0.19 0.49
AcK187 GDH		0.64 0.00	−0.42 0.08	−0.14 0.59	−0.30 0.22	−0.53 0.02	−0.63 0.01	−0.18 0.48	−0.66 0.00	0.42 0.08	−0.52 0.03	0.80 0.00	0.58 0.01	0.46 0.05	0.82 0.00		0.76 0.00	−0.07 0.77	−0.02 0.93	−0.17 0.54
AcK84 GDH		0.52 0.03	−0.50 0.04	−0.22 0.38	−0.36 0.14	−0.69 0.00	−0.73 0.00	−0.32 0.20	−0.77 0.00	0.04 0.89	−0.52 0.03	0.56 0.02	0.51 0.03	0.17 0.51	0.82 0.00	0.71 0.00		0.05 0.85	−0.23 0.35	0.07 0.79
AcK5 LDHA		−0.10 0.68	0.17 0.51	−0.02 0.92	−0.15 0.54	−0.21 0.41	−0.11 0.67	−0.11 0.68	−0.28 0.26	−0.16 0.52	−0.23 0.35	−0.08 0.75	−0.15 0.55	0.02 0.94	0.36 0.15	0.27 0.28	0.10 0.69		0.17 0.49	0.02 0.93
AcK156 LDHB		−0.06 0.82	0.38 0.12	0.07 0.78	0.20 0.43	−0.09 0.73	−0.04 0.89	0.13 0.61	−0.06 0.80	0.11 0.65	0.10 0.70	−0.24 0.34	−0.10 0.70	−0.08 0.75	0.11 0.66	0.03 0.91	0.00 0.99	0.39 0.11		0.03 0.91
AcK3 1433Z		−0.44 0.09	−0.01 0.97	−0.24 0.37	−0.09 0.74	−0.11 0.69	−0.19 0.47	−0.20 0.46	−0.11 0.68	−0.08 0.77	0.11 0.69	−0.17 0.52	−0.11 0.68	−0.48 0.06	−0.27 0.32	−0.24 0.37	−0.06 0.84	−0.37 0.16	−0.05 0.84	

**Table 2:** Correlation analysis of the metabolic role of the brain protein acetylatrion. Total acetylation of the brain proteins (AcK), SIRT3 and SIRT2 protein levels, enzymatic activities (act) related to PDHC, and redox metabolism are correlated to each other. Pearson’s correlation coefficients and *p*-values are presented in each cell of the table—above and below, respectively. The data obtained for the control rats and those which received thiamine are presented in the table parts below and above the diagonal line, correspondingly. Positive correlations are colored in red (*p* < 0.05), negative correlations are colored in blue (*p* < 0.05).

	Thiamine	AcK Total	SIRT3	SIRT2	PDHC act	OGDHC act	IDH act	GDH act	MDH act	ME act	CATA	THTM	ERO1A	PDIA1	PDIA3	PDIA6	GSHR	TRXR1	TXNL1
Control																			
AcK			−0.11 0.65	−0.43 0.07	0.20 0.44	0.20 0.42	−0.06 0.83	−0.36 0.14	−0.55 0.02	−0.31 0.21	0.53 0.03	−0.42 0.08	−0.80 0.00	−0.27 0.27	−0.38 0.12	−0.62 0.01	−0.34 0.17	−0.42 0.08	0.42 0.08
SIRT3		0.43 0.08		0.31 0.21	0.00 0.99	0.06 0.83	0.10 0.71	0.09 0.73	−0.23 0.36	0.13 0.61	−0.04 0.89	0.42 0.09	−0.04 0.88	0.22 0.38	0.41 0.09	0.24 0.33	0.18 0.48	0.28 0.26	0.25 0.32
SIRT2		−0.64 0.00	0.08 0.75		−0.21 0.41	−0.16 0.53	0.12 0.63	−0.02 0.94	0.28 0.26	0.62 0.01	−0.23 0.36	0.92 0.00	0.64 0.00	0.09 0.72	0.53 0.02	0.62 0.01	0.49 0.04	0.67 0.00	0.22 0.38
PDHC act		0.72 0.00	0.42 0.08	−0.45 0.06		0.09 0.73	−0.27 0.27	0.28 0.26	−0.25 0.32	−0.47 0.05	0.49 0.04	−0.24 0.34	−0.08 0.75	−0.25 0.31	−0.26 0.29	−0.23 0.36	−0.46 0.05	−0.30 0.23	−0.29 0.24
OGDHC act		0.72 0.00	0.18 0.46	−0.66 0.00	0.29 0.24		0.24 0.34	−0.10 0.69	−0.50 0.04	−0.52 0.03	0.15 0.56	−0.29 0.24	−0.31 0.22	0.42 0.08	0.14 0.57	−0.15 0.54	−0.05 0.86	−0.07 0.79	0.19 0.44
IDH act		0.22 0.37	−0.09 0.72	−0.53 0.02	0.33 0.18	0.15 0.56		0.09 0.73	−0.14 0.57	−0.04 0.87	−0.06 0.81	0.05 0.83	−0.18 0.48	0.14 0.59	0.04 0.88	−0.10 0.68	0.09 0.73	0.00 1.00	−0.03 0.89
GDH act		0.07 0.78	0.31 0.21	0.03 0.92	0.17 0.50	−0.05 0.85	0.22 0.38		0.00 0.99	−0.01 0.98	−0.12 0.63	0.02 0.95	0.17 0.50	0.03 0.92	−0.22 0.37	−0.04 0.87	−0.32 0.19	−0.30 0.22	−0.63 0.01
MDH act		−0.56 0.02	−0.44 0.06	0.52 0.03	−0.57 0.01	−0.47 0.05	−0.28 0.26	0.15 0.54		0.66 0.00	−0.28 0.26	0.26 0.30	0.71 0.00	0.03 0.92	0.28 0.26	0.59 0.01	0.44 0.07	0.42 0.08	−0.31 0.21
ME act		−0.58 0.01	−0.26 0.30	0.36 0.15	−0.65 0.00	−0.19 0.44	−0.31 0.21	−0.49 0.04	0.40 0.10		−0.15 0.55	0.69 0.00	0.51 0.03	−0.03 0.91	0.31 0.22	0.48 0.04	0.45 0.06	0.44 0.07	−0.05 0.85
CATA		0.63 0.00	0.62 0.01	−0.31 0.21	0.34 0.17	0.62 0.01	0.17 0.51	0.51 0.03	−0.35 0.16	−0.42 0.09		−0.31 0.21	−0.39 0.11	−0.45 0.06	−0.35 0.15	−0.52 0.03	−0.43 0.07	−0.38 0.12	0.07 0.78
THTM		−0.43 0.08	0.31 0.21	0.90 0.00	−0.30 0.23	−0.52 0.03	−0.36 0.15	0.02 0.93	0.28 0.27	0.25 0.32	−0.14 0.58		0.57 0.01	0.03 0.89	0.47 0.05	0.56 0.01	0.43 0.08	0.58 0.01	0.17 0.51
ERO1A		−0.50 0.03	−0.03 0.92	0.62 0.01	−0.28 0.26	−0.46 0.06	−0.39 0.11	0.23 0.36	0.62 0.01	0.32 0.19	−0.22 0.39	0.48 0.04		0.26 0.30	0.55 0.02	0.80 0.00	0.52 0.03	0.63 0.01	−0.25 0.32
PDIA1		−0.53 0.02	−0.04 0.89	0.14 0.59	−0.53 0.02	−0.25 0.33	−0.05 0.85	0.10 0.69	0.31 0.22	0.54 0.02	−0.12 0.63	0.15 0.54	0.48 0.04		0.76 0.00	0.55 0.02	0.69 0.00	0.56 0.02	0.02 0.93
PDIA3		−0.40 0.10	0.14 0.58	0.20 0.42	−0.39 0.11	−0.21 0.39	−0.06 0.80	−0.03 0.89	0.25 0.32	0.50 0.03	−0.13 0.60	0.25 0.31	0.58 0.01	0.87 0.00		0.85 0.00	0.90 0.00	0.92 0.00	0.28 0.26
PDIA6		−0.53 0.02	0.01 0.97	0.43 0.07	−0.48 0.04	−0.37 0.13	−0.28 0.27	0.25 0.31	0.53 0.02	0.45 0.06	−0.20 0.43	0.38 0.12	0.82 0.00	0.81 0.00	0.83 0.00		0.83 0.00	0.90 0.00	0.01 0.98
GSHR		−0.46 0.06	0.00 0.99	0.17 0.49	−0.28 0.27	−0.41 0.09	0.00 1.00	−0.18 0.48	0.18 0.47	0.46 0.05	−0.45 0.06	0.21 0.39	0.44 0.07	0.78 0.00	0.89 0.00	0.71 0.00		0.93 0.00	0.22 0.38
TRXR1		−0.57 0.01	0.00 0.99	0.37 0.13	−0.56 0.02	−0.30 0.23	−0.22 0.39	−0.03 0.92	0.45 0.06	0.63 0.00	−0.22 0.38	0.35 0.16	0.67 0.00	0.91 0.00	0.94 0.00	0.90 0.00	0.82 0.00		0.27 0.27
TXNL1		0.05 0.85	−0.23 0.36	−0.21 0.39	−0.16 0.53	0.09 0.72	0.22 0.37	−0.71 0.00	−0.12 0.63	0.40 0.10	−0.19 0.46	−0.04 0.87	−0.23 0.37	0.21 0.41	0.28 0.26	−0.10 0.69	0.32 0.20	0.20 0.42	

## Data Availability

The data presented in this study are available in this article (summarized in figures and Tables, including Supplementary data). The raw data are available on request from the corresponding author.

## Supplementary Materials

The following are available online at https://www.mdpi.com/article/10.3390/ijms22158006/s1.

## Abbreviations

PDH	pyruvate dehydrogenase
PDHC	pyruvate dehydrogenase complex
OGDH	2-oxoglutarate dehydrogenase
OGDHC	2-oxoglutarate dehydrogenase complex
GDH	glutamate dehydrogenase
MDH	malate dehydrogenase
IDH	isocitrate dehydrogenase
LDH	lactate dehydrogenase
ME	malic enzyme
ThDP	thiamine diphosphate
ANOVA	analysis of variance
TBST	Tris-buffered saline with 0.1% Tween-20
MS	mass spectrometry
LC-MS/MS	liquid chromatography with tandem mass spectrometry
LTQ	linear trap quadropole
