# Supplementary material for: Daytime Dependence of the Activity of the Rat Brain Pyruvate Dehydrogenase Corresponds to the Mitochondrial Sirtuin 3 Level and Acetylation of Brain Proteins, All Regulated by Thiamine Administration Decreasing Phosphorylation of PDHA Ser293
Source: Int J Mol Sci. 2021 Jul 27;22(15):8006. doi: 10.3390/ijms22158006 (PMC8348093; doi:10.3390/ijms22158006)
Supplement: Supplementary file 1 [file ijms-22-08006-s001.zip › ijms-1302561-supplementary.pdf]

Daytime dependence of the activity of the rat brain pyruvate dehydrogenase corresponds to the mitochondrial sirtuin 3 level and acetylation of brain proteins, all regulated by thiamine administration decreasing phosphorylation of PDHA Ser293

Vasily A. Aleshin [1,2], Artem V. Artiukhov [1,2], Thilo Kaehne [3], Anastasia V. Graf [1,4,5], Victoria I. Bunik [1,2,6,*]

1 A.N. Belozersky Institute of Physicochemical Biology, Lomonosov Moscow State University, 119991 Moscow, Russia
2 Faculty of Bioengineering and Bioinformatics, Lomonosov Moscow State University, 119991 Moscow, Russia
3 Institute of Experimental Internal Medicine, Otto-von-Guericke University, Magdeburg, Germany
4 Faculty of Nano-, Bio-, Informational, Cognitive and Socio-humanistic Sciences and Technologies at Moscow Institute of Physics and Technology, Maximova Street 4, 123098
5 Faculty of Biology, Lomonosov Moscow State University, 119234 Moscow, Russia
6 Department of Biochemistry, Sechenov University, Trubetskaya, 8, bld. 2, 119991 Moscow, Russia;
* Correspondence: bunik@belozersky.msu.ru (V.I.B.)

Supplementary Materials

Supplementary Table S1. The peptides (up to four per protein, marked as p1-p4), used for the relative quantification by MS and the forms of acetylatable peptides or the PDHA1-Ser293-containing peptide with/without phosphorylation are summarized in this table. Dependent on the number of the lysine, arginine and histidine residues in a peptide, several charged precursor variants (2+, 3+ or 4+) with monoisotopic precursor mass and the C13-isotopomepic variants ([M+1] and [M+2]) have been detected to increase the quantification accuracy. ox – Met oxidation (+16), cam – Cys carbamidomethylation (+57), da – Asn deamidation (+1), ph – Ser phosphorylation (+80), n.a. – not analyzed.

**Peptide**	**Specification**	**C13-isotopomepic variants of the precursors**		
		**monoisotopic**	**[M+1]**	**[M+2]**
VAPEEHPVLLTEAPLNPK	ACTB p1	977.5358++	978.0373++	978.5387++
		652.0263+++	652.3606+++	652.6949+++
DLYANTVLSGGTTMoxYPGIADR	ACTB p2	1116.0361++	1116.5375++	1117.0386++
QEYDESGPSIVHR	ACTB p3	758.8550++	759.3564++	759.8577++
		506.2391+++	506.5734+++	506.9076+++
PSGNYVGDSDLQLER	TBB3 p1	825.3919++	825.8934++	826.3947++
EVDEQMoxLAIQSK	TBB3 p2	703.8452++	704.3467++	704.8474++
ISVYYNEASSHK	TBB3 p3	699.3384++	699.8399++	700.3412++
FANDATFEIK	PDHA1 p1	578.2877++	578.7892++	579.2905++
		466.5614+++	466.8957+++	467.2299+++
LEEGPPVTTVLTR	PDHA1 p2	706.3932++	706.8947++	707.3960++
LPCcamIFICcamENNR	PDHA1 p3	718.3447++	718.8461++	719.3464++
YHGHSMoxSDPGVSYR	PDHA1 Ser293	804.8466++	805.3480++	805.8489++
		536.9002+++	537.2344+++	537.5683+++
YHGHSphMoxSDPGVSYR	PDHA1 Ph-Ser293	844.8298++	845.3312++	n.a.
		563.5556+++	563.8899+++	564.2238+++
VVSPWNSEDAK	PDHB p1	616.3013++	616.8028++	617.3041++
EGIECcamEVINLR	PDHB p2	666.3348++	666.8362++	667.3369++
VTGADVPMoxPYAK	PDHB p3	632.8157++	633.3172++	633.8179++

ILEDNSIPQVK	PDHB p4	628.3483++	628.8497++	629.3511++
AAPAAAAAAPPGPR	DLAT p1	594.8278++	595.3292++	595.8305++
DIDSFVPTK	DLAT p2	511.2637++	511.7652++	512.2665++
ILVPEGTR	DLAT p3	442.7636++	443.2651++	443.7664++
VFVSPLAK	DLAT p4	430.7656++	431.2671++	431.7685++
NQVTATTADGSTQVIGTK	DLD p1	896.4578++	896.9592++	897.4606++
IDVSVEAASGGK	DLD p2	566.7959++	567.2973++	567.7986++
ALTGGIAHLFK	DLD p3	564.3322++	564.8337++	565.3350++
		376.5572+++	376.8915+++	n.a.
VGKFPFAANSR	DLD p4	398.5524+++	398.8867+++	399.2209+++
EINLLPDR	PDK2 p1	485.2718++	485.7733++	486.2746++
LPVYNK	PDK2 p2	367.2158++	367.7173++	n.a.
HAVGNNEFGAVDHER	PDP1 p1	551.2572+++	551.5915+++	551.9257+++
SPSTGLYANLEK	SIRT2 p1	640.3301++	640.8315++	641.3329++
EHANIDAQSGSQASNPSATVSPR	SIRT2 p2	1162.5467++	1163.0481++	1163.5494++
		775.3669+++	775.7012+++	776.0354+++
CcamYTQNIDTLER	SIRT2 p3	706.8274++	707.3288++	707.8295++
VTQSNFAVGYKacTDEFQLHTN<br>VNDGTEFGGSIYQK	VDAC1<br>Ac-Lys174	1279.6061+++	279,9404+++	1280,2747+++
VTQSNFAVGYK	VDAC1<br>Lys174	607.3142++	607,8157++	608,3170++
VNNSSLIGLGYTQTLKPGIK	VDAC1 p1	701.7318+++	702,0661+++	702,4004+++
KLETAVNLAWTAGNSNTR	VDAC1 p2	649.3412+++	649,6755+++	650,0097+++
KacIGLFGGAGVGK	ATPB<br>Ac-Lys201	573.3375++	573,8390++	574,3403++
AHGGYSVFAGVGER	ATPB p1	703.8442++	704,3456++	704,8470++
		469.5652+++	469,8995+++	470,2337+++
FTQAGSEVSALLGR	ATPB p2	718.3806++	718,8821++	719,3834++
LVLEVAQHLGESTVR	ATPB p3	825.9623++	826,4637++	826,9651++
		550.9773+++	551,3116+++	551,6458+++
VLDSGAPIKIPVGPETLGR	ATPB p4	640.3702+++	640,7045+++	641,0388+++
ISGASEKacDIVHSGLAYTMoxER	GDH<br>Ac-Lys503	1111.5415++	1112.0430++	1112.5441++
		741.3634+++	741.6977+++	742.0318+++
ISGASEKDIVHSGLAYTMoxER	GDH<br>Lys503	1090.5362++	1091.0377++	1091.5388++
		727.3599+++	727.6942+++	728.0283+++
		545.7718++++	546.0225++++	546.2730++++
AGVKacINPK	GDH<br>Ac- Lys187	434.7662++	435.2676++	435.7689++
GASIVEDKacLVEDLKTR	GDH<br>Ac- Lys84	907.9965++	908.4980++	908.9994++
		605.6668+++	606.0011+++	606.3353+++
GASIVEDKLVEDLKTR	GDH<br>Lys84	591.6633+++	591.9976+++	592.3318+++
		443.9993++++	444.2500++++	444.5007++++
DDGSWEVIEGYR	GDH p1	713.3177++	713.8192++	714.3205++
DSNYHLLMoxSVQESLER	GDH p2	968.9571++	969.4585++	969.9595++
		646.3072+++	646.6414+++	646.9754+++
AALKacDQLIVNLLK	LDHA<br>Ac- Lys5	740.9585++	741.4600++	741.9614++
VTLTPDEEAR	LDHA p1	565.7880++	566.2895++	566.7908++
LVIITAGAR	LDHA p2	457.2951++	457.7965++	458.2979++
LSGLPKacHRVIGSGCcamNdaLD<br>SAR	LDHB<br>Ac-Lys156	727.3795+++	727.7138+++	728.0478+++

LSGLPK	LDHB Lys156	307.6972++	308.1987++	308.7000++
MoxDKacNELVQK	1433Z Ac- Lys3	581.7923++	582.2937++	582.7942++
FLIPNASQPESK	1433Z p1	665.8537++	666.3552++	666.8565++
SVTEQGAELSNEER	1433Z p2	774.8604++	775.3619++	775.8632++
VVSSIEQKTEGAEK	1433Z p3	502.2666+++	502.6009+++	502.9352+++
FNSANEDNVTQVR	CATA p1	747.3526++	747.8540++	748.3553++
FQGTQPEPR	THTM p1	530.2645++	530.7660++	531.2673++
AQPEHVISQGR	THTM p2	611.3204++	611.8218++	612.3230++
		407.8827+++	408.2169+++	408.5511+++
LGAVDESLSEETQK	ERO1A p1	753.3701++	753.8716++	754.3729++
HLLQNVH	ERO1A p2	430.7405++	431.2419++	431.7432++
		287.4961+++	287.8303+++	288.1645+++
VDATEESDLAQQYGVR	PDIA1 p1	890.9210++	891.4225++	891.9238++
NNFEGEITK	PDIA1 p2	526.2564++	526.7578++	527.2591++
YKPESDELTAEK	PDIA1 p3	705.3434++	705.8449++	706.3462++
		470.5647+++	470.8990+++	471.2332+++
DLFSDGHSEFLK	PDIA3 p1	697.8330++	698.3345++	698.8358++
		465.5577+++	465.8921+++	466.2263+++
IFRDGEEAGAYDGPR	PDIA3 p2	826.8868++	827.3882++	827.8896++
		551.5936+++	551.9279+++	552.2621+++
LAPEYEAAATR	PDIA3 p3	596.3039++	596.8053++	597.3066++
LAAVDATVNQVLASR	PDIA6 p1	764.4281++	764.9295++	765.4309++
GSTAPVGGGSFPNITPR	PDIA6 p2	807.9154++	808.4168++	808.9181++
GESPVDYDGGR	PDIA6 p3	576.2518++	576.7532++	577.2545++
LGIQTDDKGHILVDEFQNTNVK	GSHR p1	1242.6401++	1243.1415++	1243.6429++
		828.7625+++	829.0968+++	829.4310+++
ALLTPVAIAAGR	GSHR p2	576.8586++	577.3600++	577.8614++
IEQIEAGTPGR	TRXR1 p1	585.8093++	586.3107++	586.8120++
TIGLETVGVK	TXNR1 p2	508.8030++	509.3044++	509.8058++
IKQHLENDPGSNEDTDIPK	TXNL1 p1	1075.5216++	1076.0231++	1076.5244++
		717.3502+++	717.6845+++	718.0187+++
VRIDQYQGADAVGLEEK	TXNL1 p2	945.9814++	946.4829++	946.9842++
		630.9900+++	631.3243+++	631.6586+++
QHLENDPGSNEDTDIPK	TXNL1 p3	954.9321++	955.4336++	955.9349++
		636.9572+++	637.2915+++	637.6257+++

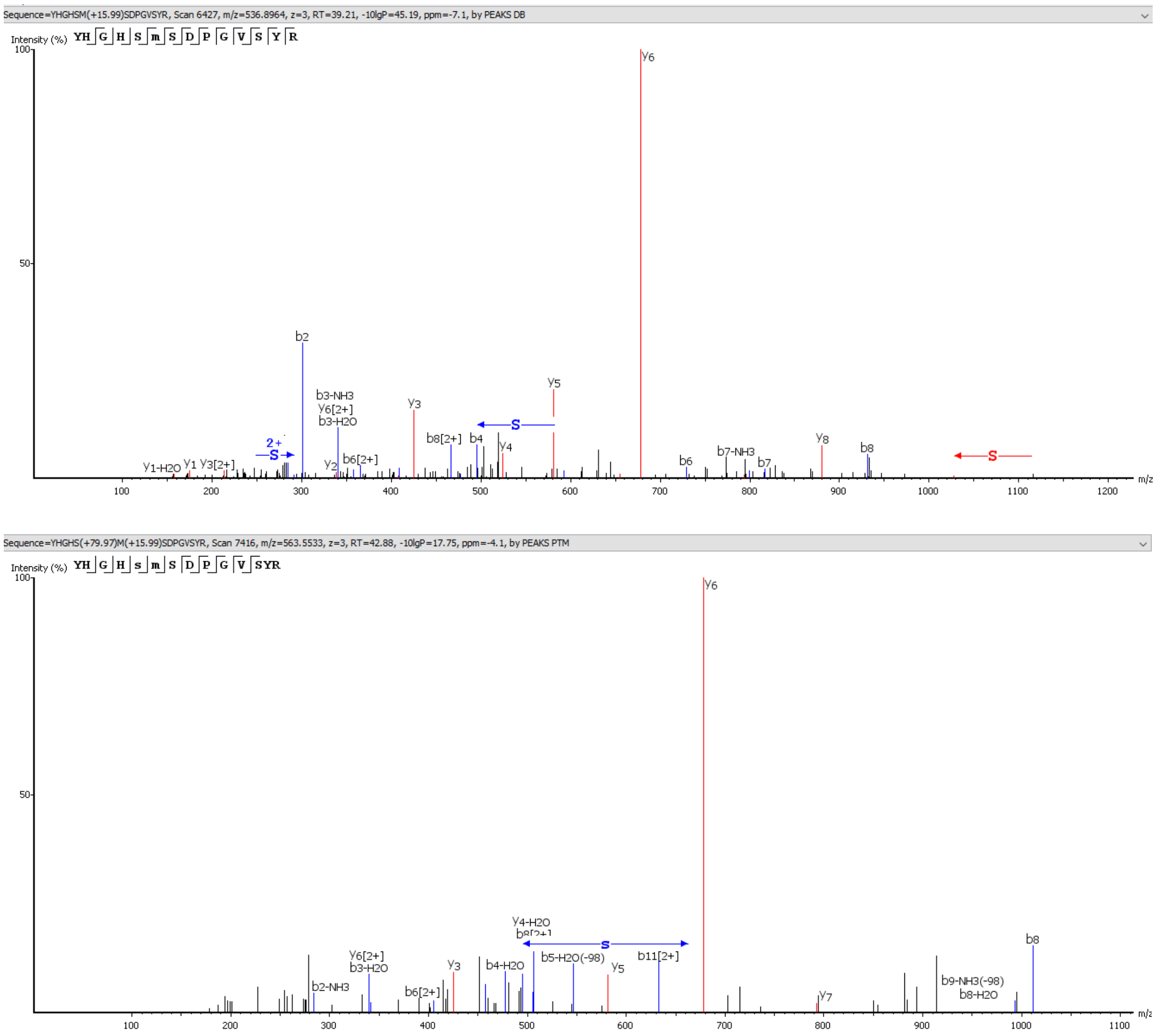

Supplementary Figure S1. Representative MS/MS spectra of the PDHA1 peptides containing dephosphorylated and phosphorylated Ser293 in the rat cerebral cortex homogenates. The sequence and parameters of the peptides are shown on the graphs. The images of the spectra were prepared in PEAKS Studio 8.0. The blue and red arrows represent the parts of the spectra, corresponding to the phosphorylatable Ser293 residue.

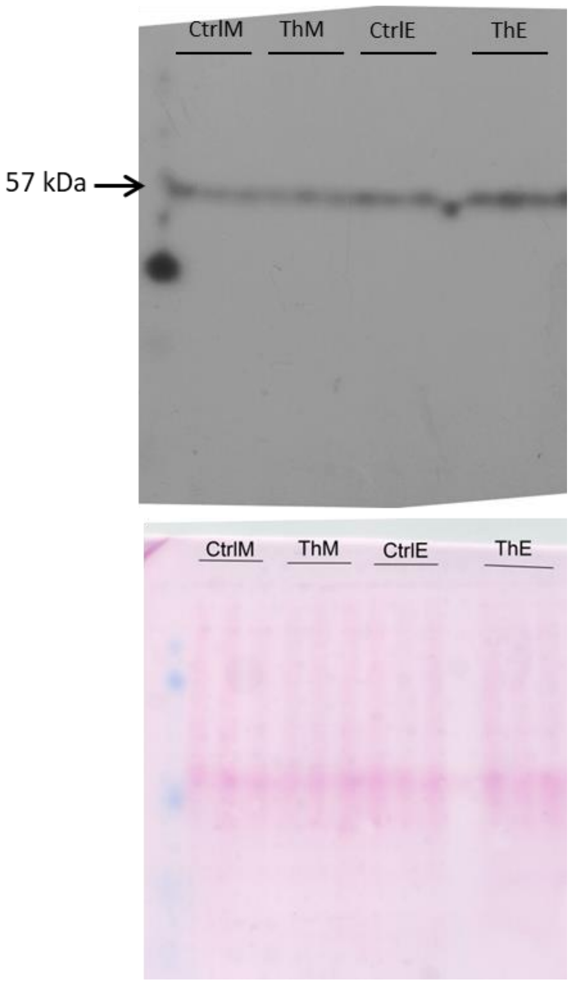

Supplementary Figure S2. Representative images of western blots for acetylated lysine residues with corresponding Ponceau S staining. Each of the lanes represents different animals from the following groups: CtrlM – control, morning (n=3); ThM – thiamine, morning (n=3); CtrlE – control, evening (n=3); ThE – thiamine, evening (n=3).

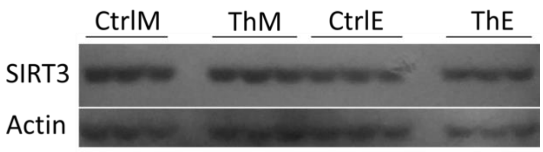

Supplementary Figure S3. Representative images of western blots for SIRT3 and Actin. Each lane correspond to different animals from the following groups: CtrlM – control, morning (n=3); ThM – thiamine, morning (n=3); CtrlE – control, evening (n=3); ThE – thiamine, evening (n=3).
